# Intratumor Heterogeneity and Therapy Resistance: Contributions of Dormancy, Apoptosis Reversal (Anastasis) and Cell Fusion to Disease Recurrence

**DOI:** 10.3390/ijms21041308

**Published:** 2020-02-15

**Authors:** Razmik Mirzayans, David Murray

**Affiliations:** Department of Oncology, Cross Cancer Institute, University of Alberta, Edmonton, AB T6G 1Z2, Canada; david.murray5@ahs.ca

**Keywords:** cancer therapy, cell fusion, dormancy, polyploid giant cancer cells, senescence, persister, apoptosis, anastasis, colony formation assay, high-throughput assays

## Abstract

A major challenge in treating cancer is posed by intratumor heterogeneity, with different sub-populations of cancer cells within the same tumor exhibiting therapy resistance through different biological processes. These include therapy-induced dormancy (durable proliferation arrest through, e.g., polyploidy, multinucleation, or senescence), apoptosis reversal (anastasis), and cell fusion. Unfortunately, such responses are often overlooked or misinterpreted as “death” in commonly used preclinical assays, including the in vitro colony-forming assay and multiwell plate “viability” or “cytotoxicity” assays. Although these assays predominantly determine the ability of a test agent to convert dangerous (proliferating) cancer cells to potentially even more dangerous (dormant) cancer cells, the results are often assumed to reflect loss of cancer cell viability (death). In this article we briefly discuss the dark sides of dormancy, apoptosis, and cell fusion in cancer therapy, and underscore the danger of relying on short-term preclinical assays that generate population-based data averaged over a large number of cells. Unveiling the molecular events that underlie intratumor heterogeneity together with more appropriate experimental design and data interpretation will hopefully lead to clinically relevant strategies for treating recurrent/metastatic disease, which remains a major global health issue despite extensive research over the past half century.

## 1. Introduction

“…we have come full circle, beginning in a period when vast amounts of cancer research data yielded little insight into underlying mechanisms to a period (1980–2000) when a flurry of molecular and genetic research gave hope that cancer really could be understood through simple and logical reductionist thinking, and finally to our current dilemma” (R.A. Weinberg [1]).

Despite Herculean efforts and the spending of billions of dollars on anticancer drug discovery and development studies for decades, cancer is currently the leading cause of death in wealthy countries. In 2018, cancer led to the deaths of over 9 million people worldwide, most of which were due to metastatic tumor burden [2]. This review addresses two reasons why metastatic disease remains largely incurable: (i) misinformation perpetrated by the misguided use of cell-based radiosensitivity and chemosensitivity assays in general, and of high-throughput multiwell plate colorimetric/fluorometric assays in particular; and (ii) intratumor heterogeneity of solid tumors with respect to metastasis and therapy resistance.

Multiwell plate assays, which continue to be widely used in anticancer agent-related studies (e.g., the NCI-60 Human Tumor Cell Line Screen) [3,4,5,6], are short-term tests (48 h drug treatment) that were developed during the aforementioned 1980–2000 period referred to by Weinberg. They were originally described to assess inhibition of proliferation, which provides a combined measure of cytostatic and cytotoxic responses, in cancer cell lines following chemotherapeutic drug treatment [7,8]. Accordingly, the NCI anticancer drug screen identifies agents capable of inhibiting proliferation in a well-characterized panel of 60 cancer cell lines [6]. Unfortunately, most authors and assay manufacturers (e.g., [9,10]) have interpreted the results obtained by such assays based on a rather simplistic, two-arm model of the DNA damage response: repair and survive (viability) or die through apoptosis (loss of viability). This simplistic model fails to account for treatment-induced proliferation arrest.

**Figure 1:**
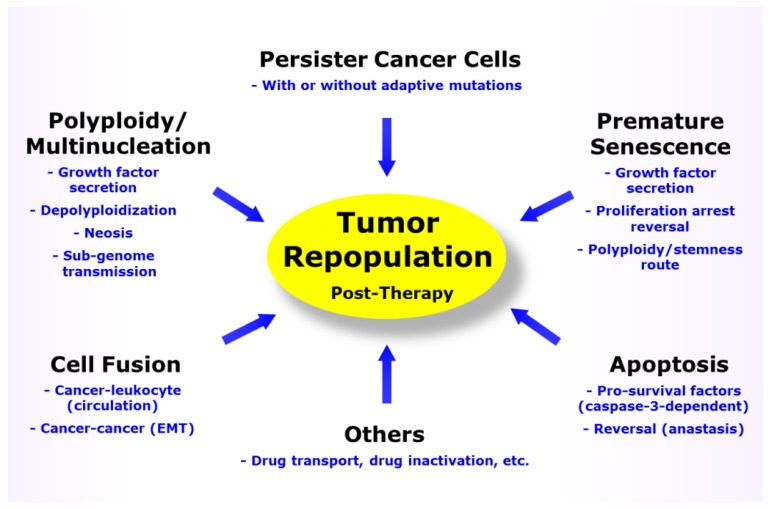
Responses contributing to solid tumor repopulation following treatment with anticancer agents. EMT, epithelial to mesenchymal transition.

A growing body of recent research indicates that acquired resistance of cancer cells to therapeutic agents is multifactorial, with several unrelated mechanisms employed simultaneously by different subsets of cancer cells within the same tumor (Figure 1). These include therapy-induced dormancy (durable proliferation arrest), paradoxically apoptotic “death” which can be reversible in solid tumor cells, and cell fusion. Such intratumor heterogeneity is not taken into account in most preclinical assays such as those performed in a multiwell plate format.

In this article, we briefly discuss the degree of complexity of the biological consequences of DNA damage in solid tumors/tumor-derived cell lines, focusing on the dark sides of dormancy, apoptosis, and cell fusion in the context of cancer therapy. In addition, we highlight the fact that the various multiwell plate cell “viability” and “cytotoxicity” assays predominantly (if not exclusively) measure cancer cell proliferation arrest (and not death) following treatment with genotoxic agents, unless the experiments are performed with non-proliferating (dormant) cultures, in which case the end point measured would most probably reflect loss of viability (death). Stated differently, while multiwell plate assays might generate misleading information with proliferating cultures treated with genotoxic agents, they may be particularly useful for identifying agents capable of killing dormant cancer cells.

## 2. Metastasis and Tumor Repopulation Associated with Cancer Cells That Might Be Overlooked or Scored as “Dead” in Preclinical Assays

For decades, a widely held tenet of oncology has been that the cellular response to DNA-damaging agents may involve activation of cell cycle checkpoints, commencement of transcriptional programs, and execution of DNA repair to promote cell survival, or when the damage is severe, initiation of apoptotic signaling to result in the deletion of injured cells. Accordingly, activation of caspase-3, the key executioner caspase in the apoptotic cascade, has been widely used as a surrogate molecular marker of cell death (e.g., [9,10,11,12,13,14]). As mentioned above, this two-armed model of the DNA damage response—repair and survive or die through apoptosis—has led to the development of numerous short-term colorimetric/fluorometric assays that are ideal for high-throughput studies. These assays are typically performed within 2–3 days after exposure to ionizing radiation or continuous drug treatment.

In view of their relative ease of performance and use of an automated multiwell plate reader to generate the colorimetric/fluorometric data, such short-term assays have become more popular for radiosensitivity/chemosensitivity assessment than the conventional long-term colony formation (or clonogenic) assay. The latter is an in vitro cell survival assay which is based on the ability of individual cells to form macroscopic colonies (each consisting of aggregates of at least 50 cells) in the time span of about two weeks after seeding the cells in tissue culture plates.

As discussed by Brown and Wouters two decades ago [15], in addition to early apoptosis, genotoxic stress also triggers a sustained proliferation-arrested response that would be largely overlooked in short-term assays, but would be detected in the colony formation assay. The proliferation-arrested response triggered by genotoxic stress was proposed to lead to cell death through secondary apoptosis and necrosis, and was called “reproductive death” [15]. Since then, the list of stress-induced responses that contribute to loss of clonogenic potential of cancer cells has expanded to include necroptosis, ferroptosis, senescence (a durable form of proliferation arrest), and mitotic catastrophe (aberrant mitosis resulting in the development of giant cells with nuclear abnormalities, including multiple nuclei) [16,17]. 

Cancer cells exhibiting any of these responses are assumed to be rapidly eliminated by different means in a tissue culture setting (e.g., cells undergoing mitotic catastrophe might be eliminated through delayed necrosis, secondary apoptosis, or an apoptosis-like death [18]), and, additionally, by the multi-step process of dying-cell clearance in vivo [17]. Based on such assumptions, the colony forming assay would still be a reliable tool for assessing cancer cell death following treatment with genotoxic agents. However, as pointed out below, such assumptions are not entirely tenable, at least for solid tumors and solid tumor-derived cell lines.

A shortcoming of preclinical assays that are widely used in anticancer drug discovery endeavors is the reliance on cell population-based analyses averaged over thousands or millions of cells. These include not only multiwell plate assays, but also immunoblotting for assessment of the status of death-related factors (e.g., caspase-3 activation for apoptosis). Time-lapse microscopy and other single-cell observation methods have revealed the coexistence of different sub-populations of malignant cells, with differing biological behaviors, within the same tumor (i.e., intratumor heterogeneity) (see Section 3). Such studies have demonstrated that cancer cell resistance to therapeutic agents, and hence disease relapse post-therapy, is largely associated with dormant cancer cells within a solid tumor/tumor-derived cell line. Thus, cancer cells exhibiting “reproductive death” (i.e., entering a dormant state) can remain viable for extended periods (weeks and months) following treatment with genotoxic agents, secrete growth-promoting factors, and give rise to therapy resistant progeny. Therapy-induced responses that contribute to tumor dormancy include (but are not limited to) polyploidy/multinucleation (Section 3.1) and a senescence-like proliferation arrest (premature senescence) (Section 3.2). Like dead cancer cells, dormant cancer cells do not form macroscopic colonies in the standard colony formation assay; however, they may be far from dead depending on context. 

In addition to dormancy, in the past decade other stress-induced responses have been identified that promote tumor repopulation post-therapy and yet might not be reflected in conventional pre-clinical assays. These include the oncogenic effect of engaging apoptotic signaling (e.g., caspase-3 activation) (Section 4.1), recovery of apoptotic cells from the brink of death (Section 4.2), and cancer cell fusion (Section 5).

After a brief discussion on the contributions that these responses make to metastasis and therapy resistance, we will further examine the endpoints measured by multiwell plate assays (e.g., crystal violet “cytotoxicity”) to illustrate that, although such assays lack specificity when performed with proliferating cultures, they may be useful for identifying agents capable of destroying dormant cancer cells (Section 7). 

## 3. Therapy-Induced Cancer Cell Dormancy and Disease Relapse

### 3.1. Multinucleated/Polyploid Cancer Cells

Of the many types of cancer cell misbehavior, dormancy, a type of active sleep, presents a major obstacle in the medical treatment of malignant solid tumors [19,20,21,22]. A subset of dormant cells are considered “giants” and contain either multiple nuclei or a highly enlarged nucleus (sometimes >100 fold) when compared to the nucleus of the bulk of cancer cells within the same tumor [23,24,25,26,27,28,29,30]. Such cells are referred to as polyploid giant cancer cells (PGCCs). PGCCs are present in most solid tumors/tumor-derived cell lines and their frequency increases following exposure to therapeutic agents. The formation of PGCCs following treatment with anticancer agents is not an infrequent response [25,26,27,28,29,30], and their frequency can be influenced by heterogeneous environment and chemotherapeutic drug gradient, increasing remarkably at high drug concentrations [25]. Given that PGCCs cease to proliferate or proliferate at a very slow rate, they are often overlooked or misrepresented as “dead” in the conventional pre-clinical assays that are performed with cultured cells or animal models (reviewed in [31,32]). However, over 60 years ago Puck and Marcus [33] reported that PGCCs (originally called giant cells), arising in cervical carcinoma (HeLa) cell cultures following exposure to ionizing radiation, remain viable and secrete growth-promoting factors. In addition to secreting pro-survival factors, PGCCs are now known to be capable of giving rise to therapy-resistant and tumor repopulating cells through (i) nuclear budding or bursting, similar to simple organisms such as fungi [34,35,36,37,38,39,40,41,42,43,44,45,46,47]; (ii) depolyploidization, involving key mediators of meiosis, self-renewal, and mitosis [48,49,50,51,52]; and (iii) horizontal transfer of their nuclear material that contain stem-cell markers to neighboring cells [53]. Consistent with these properties, a significant number of recent reports with different biological systems (e.g., tumor-derived cell lines, animal models, and specimens from cancer patients) have underscored the key roles played by PGCCs in tumorigenesis, metastasis, and disease relapse after conventional cancer treatments. The contributions that PGCCs make to therapy resistance is demonstrated for various types of solid tumors, including ovarian cancer [43,44], prostate cancer [23,47], brain cancer [26,28], renal cancer [54], colon cancer [46], and breast cancer [45,55], including the most aggressive form (triple negative) [55] for which chemotherapy remains the cornerstone therapeutic. There have been several reviews on the formation, stemness, and tumorigenicity of PGCCs before and after exposure to cancer therapeutic agents (e.g., [56,57,58,59]).

The complex life cycle experienced by PGCCs that can result in the emergence of therapy resistant progeny is consistent with the atavistic model of cancer [60,61,62,63,64,65,66,67,68,69]. In this model, “cancer is interpreted as a reversion to phylogenetically prior capabilities, namely the release of a highly conserved survival program encrypted in every eukaryotic cell, and hence in every multicellular animal. This ancient program would have evolved during the pre-Cambrian period, when the selective forces acting on unicellular organisms were favoring adaptations prioritizing the continuation/proliferation of cellular life in different, often adverse, environmental conditions” [64]. 

### 3.2. Senescent Cancer Cells

Loss of wild-type p53 function is permissive for the emergence of PGCCs. In p53 wild-type cells, on the other hand, therapy-induced dormancy is largely (but not entirely) associated with a senescence-like state called stress-induced premature senescence (SIPS) or therapy-induced cellular senescence [70,71,72,73,74,75,76]. Characteristic features of this state include the acquisition of enlarged and flattened cell morphology, presence of *β*-galactosidase activity at suboptimal conditions (i.e., pH 6), absence of cell division, apoptosis resistance, and maintenance of cell viability and metabolic activity. A key molecular mediator of SIPS is the cyclin-dependent kinase (CDK) inhibitor p21^WAF1^ (p21), which functions downstream in the p53 signaling pathway [75,77]. Although widely cited for its ability to temporarily halt cell-cycle progression under stressful conditions, p21 has emerged as a multifunctional protein capable of downregulating p53, suppressing apoptosis by acting at different levels of the death cascade (e.g., inhibiting caspase-3), and actively participating in the regulation of genes involved in growth arrest and senescence (reviewed in [77]). Furthermore, p21 forms a positive feedback loop with ATM, and this interaction has been shown to underlie the apoptosis resistance of cancer cells that have undergone SIPS following treatment with chemotherapeutic agents [78]. Cancer cells lacking wild-type p53 activity can also undergo SIPS through p21-dependent [79,80] and independent mechanisms [81]. In addition to p21, CDK inhibitors that can drive SIPS include p16^INK4a^, p15^INK4b^, and p27^KIP1^ [80,81,82]. 

Cancer cells in the senescence-like state secrete a myriad of biologically active factors that affect surrounding cells by activating various cell surface receptors and corresponding signal transduction pathways [83,84]. The beneficial and harmful consequences of this so-called “senescence-associated secretory phenotype” (SASP) has been the subject of much discussion (e.g., [73,83,84]). In addition to detrimental effects of SASP in the context of cancer therapy, the proliferation-arrested state in solid tumor cells that have undergone SIPS is often reversible, leading to disease relapse [82,85,86]. Furthermore, under some conditions, depending on cell type and level/type of genotoxic stress, solid tumor cells undergoing SIPS can become multinucleated and/or polyploid, entering the PGCC-stemness-tumor repopulation cycle [41,85,86,87,88]. In short, therapy-induced dormancy through SIPS is now accepted to represent a long-term survival mechanism for most solid tumors. Accordingly, these observations have led to a two-step anticancer therapeutic concept, with the first step of inevitable senescence-inducing radiotherapy/chemotherapy being followed by “senotherapy” to trigger senescent cancer cell lysis before they will have the opportunity to exert their harmful effects [74].

To this end, Crescenzi et al. [78] reported that downregulating either p21 or ATM in MCF7 (breast carcinoma) and A549 (lung carcinoma) cells that had undergone SIPS following exposure to the chemotherapeutic drug doxorubicin resulted in their loss of viability. Similarly, Hsu et al. [75] recently reported that A549 cells that had undergone SIPS in response to doxorubicin treatment could be targeted by Navitoclax (ABT-263), an inhibitor of anti-apoptotic proteins, to induce loss of viability. (In both studies, the fate of non-proliferating cells that have undergone SIPS was determined using a multiwell plate “viability” assay. The end points measured under these conditions are outlined in Section 7.3.)

### 3.3. Anti-Proliferative Drug-Tolerant “Persister” Cancer Cells 

In addition to PGCCs and senescent cancer cells, which typically exhibit a highly enlarged morphology, some cancer cells in solid tumors are indistinguishable from the bulk of cancer cells with respect to morphology, but show selective tolerance towards antiproliferative cancer drugs merely as a result of their dormant state or very slow proliferation rate [89,90,91]. These cells are called drug-tolerant “persister” cells, borrowing terminology from microbiology, where persisters are non-growing or slow-growing bacteria with the ability to survive antibiotic treatment (reviewed in [22,92]. Dormant persister cancer cells similarly survive concentrations of antiproliferative drugs that are effective against the proliferating bulk population. After removal of the drug, a period referred to as drug “holidays”, dormant persister cells resume proliferation and regrow into a new population that is as sensitive to antiproliferative drugs as the bulk population [22]. The reversibility of dormancy of persister cells observed during drug holidays is attributed to an epigenetic rather than a genetic mechanism [22,92]. 

### 3.4. Adaptive Mutation in Persister Cells

In microorganisms, hostile environments such as the presence of antibiotics trigger adaptive mutations in persister cells, resulting in their growth in the new environment [93,94,95,96]. Once the adaptation in the new landscape is established, the mutator phenotype is suppressed to prevent further accumulation of mutations and increased mutational load [96,97,98,99,100,101]. These events are controlled by epigenetic mechanisms [22,92]. In the early 2000s, it was reported that hypoxic stress of human cancer cells also leads to their acquired resistance to low oxygen levels through adaptive mutations [102,103,104]. Recently, Russo and colleagues [91] provided evidence suggesting that colon cancer patients undergoing targeted therapy by inhibitors of EGFR (epidermal growth factor receptor) and/or BRAF (serine/threonine-protein kinase B-Raf) might also become resistant to these treatments by a similar mechanism. The authors demonstrated that:

➢ Treatment of colon cancer cells with targeted therapies decreased their mismatch-repair (MMR) and homologous-recombination proficiency, resulting in their increased dependency on error-prone DNA repair.
➢ Patient-derived tumor samples underwent a similar response, with samples from colorectal-cancer residual disease exhibiting downregulated MMR proteins.
➢ Markers of DNA damage were elevated in colorectal-cancer cells treated with targeted therapies.
➢ Levels of reactive oxygen species were concomitantly increased in these cells, which could account for the accumulation of DNA damage.
➢ Treatment with targeted therapies triggered initiation of a stress-response program, leading to adaptive mutability and genetic instability, rather than simply exerting nonspecific effects that caused DNA damage.
➢ As seen in adaptive mutability of microorganisms, the changes in DNA repair seen in cancer cells following treatment with targeted therapeutic drugs was transient and reverted to pre-treatment status once growth capability in the presence of the drugs was restored.

These observations led Russo et al. [91] to conclude that “in cells of multicellular organisms, stress-induced mutagenesis is not operational. However, in cancer cells that have lost tissue-imposed homeostasis, and in many ways operate like unicellular organisms, this ancestral program is still available and is unleashed by oncoprotein-targeted drugs.”

These observations also demonstrate that, while the efficient operation of the high-fidelity DNA repair machinery is fundamental for maintaining genome stability and homeostasis of non-cancerous cells, their downregulation in cancer cells provides a survival mechanism through genome instability and adaptive mutations. It is noteworthy that a subset of human solid tumor-derived cell lines exhibit high levels of spontaneous DNA double-strand breaks; these include cell lines that lack p53 (e.g., HCT116p53−/− colon carcinoma; SKOV3 ovarian carcinoma) or p21 (e.g., HCT116p21−/− colon carcinoma), and those expressing mutant p53 (e.g., MDA-MB-231 breast carcinoma) [105]. 

## 4. Therapy-Induced Cancer Cell Apoptosis and Disease Relapse

Genotoxic anticancer agents activate the mitochondrial or intrinsic pathway of apoptosis (reviewed in [106,107,108]). The process is initiated by mitochondrial outer membrane permeabilization (MOMP), resulting in the release of cytochrome c and other intermembrane space proteins. Cytochrome c binds to apoptosis-activating factor-1 (APAF-1) and caspase-9, forming the apoptosome. This interaction activates caspase-9, which then activates the executioner caspases (caspase-3 and caspase-7) and deoxyribonuclease (DNase) activity downstream. The execution phase leading to cell demise includes the externalization of phosphatidylserine (PS) on the outer plasma membrane leaflet, nuclear translocation of a caspase-activated DNase that leads to internucleosomal DNA cleavage, nuclear condensation, cell shrinkage, and the eventual formation of apoptotic bodies. Stress-induced MOMP was originally assumed to inevitably commit a cell to death and was therefore considered a point of no return [109,110,111]. Accordingly, multiple biochemical and morphologic assays have been used—either singly or in combination—to assess apoptotic cell death. The standard apoptotic assays measure MOMP status, caspase activation, PS externalization (Annexin V staining), nuclear fragmentation (e.g., TUNEL staining; flow cytometric measurement of cells with sub-G1 DNA content), and microscopic assessment of cell morphology. In the past decade, however, the dark side of apoptosis in the context of cancer therapy has been demonstrated by two series of counterintuitive observations: functions of caspases outside of apoptosis that promote tumor repopulation, and recovery of apoptotic cells from the brink of death.

### 4.1. Role of Caspase-3 in Tumor Repopulation

Although MOMP and caspase-3 activation continue to be used as molecular markers of cell death, relatively recent studies demonstrated that, under some circumstances, instead of functioning as an apoptosis executioner, active caspase-3 plays an important role in carcinogenesis, metastasis, and therapy resistance (reviewed in [107,112,113,114,115,116,117,118,119,120]). Caspase-3 can stimulate tumor cell growth through various routes. One route involves caspase-3-mediated secretion of pro-survival factors such as prostaglandin E_2_ that promote enrichment of tumor repopulating cells [115,116,117,118,119]. Another route is through caspase-3/DNase-mediated accumulation of genome instability (e.g., DNA double-strand breaks) that trigger ATM-dependent activation of the transcription factors NF-κB and STAT3, known drivers of tumor growth [107,120]. In addition to secreting pro-survival factors, apoptotic cells release exosomes (nano-sized lipid vesicles containing proteins, nucleic acids, micro RNAs, etc.) that maintain the viability of the neighboring cells [107,114,121]. Caspase 3 appears to play a role in a late phase of exosome biosynthesis, such as cleavage of the pore-forming protein DFNA5 [121]. Thus, the point of no return for apoptotic cell death must be downstream of MOMP and caspase activation.

### 4.2. Recovery of Apoptotic Cancer Cells from the Brink of Death

A decade ago, Tang and associates [122] reported studies that were designed to determine whether caspase activation represents a point of no return. Apoptosis was triggered in the HeLa cervical carcinoma cell line after treatment with Jasplakinolide (a cytotoxic natural product), staurosporine (a non-selective inhibitor of protein kinases), and ethanol. As expected, incubation of HeLa cells with these agents induced caspase activation, mitochondrial fragmentation, nuclear condensation, and cell shrinkage. Surprisingly, these cellular and biochemical markers of apoptosis vanished within 24 h after removal of the stimuli. This recovery phenomenon that rescues cells from the brink of death is referred to as anastasis [108,123,124], and has been reported by multiple groups for a wide range of human solid tumor-derived cell lines after treatment with ethanol or chemotherapeutic drugs [122,123,124,125,126,127,128,129,130,131,132,133,134,135,136,137,138,139,140,141,142,143,144,145,146,147,148,149,150,151,152,153,154,155]. The important apoptotic checkpoints that cancer cells need to negotiate to continue to survive and proliferate include not only cytochrome c release, caspase activation, DNA damage, nuclear condensation, and fragmentation, but also apoptotic body formation [123]. Time-lapse live cell microscopy has demonstrated that cancer cell fragments resulting from the formation of apoptotic bodies can coalesce to give rise to cells with apparently normal morphology, often displaying an increased number of micronuclei and chromosomal abnormalities that can lead to increased aneuploidy [125,127]. Consistent with these observations, studies have revealed that anastatic cancer cells acquire stem cell-like properties that can promote tumor progression, therapy resistance, and disease recurrence [125,126,127,130].

Seervi and associates studied the reversibility of late-stage apoptosis in relation to the extent of MOMP in human cervical (HeLa) and breast (MDA-MB-231) cancer cell lines after treatment with the chemotherapeutic drugs paclitaxel and etoposide [132]. Late stage apoptosis was verified by positive staining with both Annexin V and propidium iodide (PI). Anastasis was observed after “limited” MOMP, when only a fraction of the mitochondria in a cell were permeabilized and the majority remained intact, as well as after “widespread” MOMP, when most of the mitochondria in a cell were permeabilized. These authors also reported comparative proteomic and transcriptomic studies suggesting a role for the nuclear-export pathway in the anastatic process of cancer cells. Furthermore, chemical inhibition of the nuclear export factor CRM1 (chromosomal region maintenance 1) effectively reduced the augmented migration and invasive capabilities of anastatic cancer cells acquired during the recovery process. These and related observations led the authors to propose that “inhibition of anastasis through the nuclear export pathway may be a potential therapeutic strategy for targeting drug-resistance, metastasis and recurrence problems during cancer treatment” [132].

### 4.3. Apoptotic Threshold

To induce widespread MOMP, Seervi et al. [132] treated cancer cells with very high concentrations of chemotherapeutic drugs for long times. For example, MDA-MB-231 cells were incubated with 200 nM paclitaxel for 24–48 h, depending on the apoptotic assay. Previously, we reported that a much lower concentration of paclitaxel was required to induce proliferation arrest in 50% of MDA-MB-231 cells (IC_50_, <2 nM) when determined by direct cell counting [133]. In our published work the cells were incubated continuously with paclitaxel for 72 h; similar results were obtained in a 72-h proliferation assay (determined by cell counting) when the cells were treated with the drug for 48 or 24 h (plus incubation in drug-free medium for 24 or 48 h, respectively) (unpublished observations). Thus, there is a remarkable discrepancy (≈100 fold) between the stress levels required to induce proliferation arrest versus apoptosis, indicating the presence of a threshold effect for apoptosis. Such discrepancy has been reported for virtually all genotoxic agents, including ionizing radiation and chemotherapeutic drugs such as cisplatin and oxaliplatin, and for various solid tumor-derived cell lines with differing p53 status (reviewed in [77,134,135]). These observations are not surprising given that relatively low levels of injury to the nuclear material are sufficient to trigger proliferation arrest, whereas very high levels of stress are required to induce the widespread MOMP necessary to initiate the intrinsic apoptotic pathway.

## 5. The Role of Cancer Cell Fusion in Metastasis and Therapy Resistance

Cancer cell fusion has emerged as a fundamental non-genetic mechanism that contributes to intratumor heterogeneity, resulting in tumor progression (invasive growth and metastasis) and therapy resistance. As recently pointed out by different authors [136,137,138,139], the potential role of fusion between cancer cells and motile leukocytes in promoting metastasis was proposed by the German pathologist Prof. Otto Aichel nearly a century ago. This intriguing hypothesis remained controversial during the 20th century when the somatic mutation theory of carcinogenesis was the dominant force driving cancer research. In the past decade, however, a significant number of reports have underscored the importance of cell fusion in tumor progression and therapy resistance in a wide range of malignancies (e.g., [136,137,138,139,140,141,142,143,144,145]). In a recent study reported by Gast and associates [144], for example, cancer cell-leukocyte hybrids were detected in peripheral blood of cancer patients, and the numbers of such hybrids in the patient’s blood correlated with disease stage and predicted overall survival. In addition, cancer cells obtained from metastatic tumors of women who years earlier had received a bone marrow transplant from a male donor were shown to carry a Y chromosome, indicating that metastasis originated from fusion between patient cancer cells and donor bone marrow cells [144]. In that study, the Y chromosome was present in metastatic cancers of all patients that were examined, which involved women with kidney, head and neck, lung, and pancreatic cancers [144]. 

As noted by those authors, the presence of tumor cells with acquired leukocyte phenotypes “supports a cell fusion mechanism in the propagation of intratumor heterogeneity, introduces a functionally significant aspect of tumor progression and evolution, identifies an unappreciated circulating hybrid cell population, and uncovers a new area of tumor cell biology” [144].

In addition to cancer cell–leukocyte fusion, fusion between cancer and cancer cells [28,146,147], between cancer and stem cells [148,149,150,151], and between cancer and stromal cells [152,153] are also known to promote tumor progression/therapy resistance. Fusion between different cell types within a solid tumor is perhaps not unexpected given that “a tumor tissue resembles chronically inflamed tissue, that chronically inflamed tumor tissue efficiently recruits macrophages, mesenchymal stem cells and hematopoietic stem/progenitor cells, that chronic inflammatory conditions are a positive trigger for cell fusion” [145].

Fused cells undergo heterokaryon-to-synkaryon transition, reflecting nuclear fusion, which results in a loss and/or re-sorting of chromosomes in a random manner [145]. These events give rise to unique hybrid cells in which the degree of aneuploidy/genomic instability is further enhanced during subsequent rounds of cell division. Fused cells and most (but not all) [154] of their hybrid descendants exhibit increased resistance to anticancer agents relative to their unfused parental cells when assessed in vitro (e.g., using flow cytometric approaches) [154] and in tumor growth studies with live animals [146,154].

Fusions can occur between several cells simultaneously within a tumor, causing the formation of PGCCs, which underlie therapy resistance, metastasis, and disease recurrence as outlined above (Section 3.1). In human glioblastoma cell cultures, for example, the formation of PGCCs following exposure to ionizing radiation has been reported to be largely associated with homotypic cell fusion [28].

## 6. Can Cancer Recurrence Be Prevented? 

In view of the disappointing current statistics on cancer-associated deaths, it must be concluded that the promises made over the decades in regard to combating this devastating disease have not become a reality. These promises included targeting cell cycle checkpoints and DNA repair machineries, signaling pathways associated with oncogenes and tumor suppressor genes, and numerous other genetic and epigenetic factors. Thus, as cancer is an extremely complex disease, its prevention has proven to be equally complex, extending far beyond modulating the DNA damage response and/or cell death pathways. It is becoming increasingly evident that, to realize the monumental goal of combating cancer, one must take into account not only the mutational basis of intrinsic/acquired therapy resistance of cancer cells, but also non-mutational events such as cancer cell dormancy, horizontal (cell-to-cell) transfer of genetic material containing cancer stem cell markers [53], an atavistic process whereby cancer cells re-express ancestral traits that includes severe stress resistance [60,61,62,63,64,65,66,67,68,69], and cancer cell fusion (e.g., cancer cell–leukocyte [28,144]) which facilitates cancer cell circulation and metastasis. Irrespective of this complexity, one must hope that a better understanding of the multifactorial nature of cancer cell resistance to therapeutic agents will finally lead to novel strategies to prevent, or at least significantly suppress, cancer recurrence. 

A step towards reaching this goal will be to understand not only the bright sides of different stress-induced responses (e.g., senescence) that might impact on the initial tumor control, but also their dark sides that might contribute to tumor repopulation. In addition, as cautioned by the Nomenclature Committee on Cell Death [155] and others [32,156,157], it is crucial to use the available tools (e.g., cell-based assays) in the right contexts to avoid generating misleading information. To this end, as discussed below, the colony formation and multiwell plate colorimetric/fluorometric assays predominantly determine the ability of a genotoxic agent to convert “dangerous” (proliferating) cancer cells to “even more dangerous” (e.g., dormant) cancer cells, rather than dead cancer cells.

## 7. Preclinical Studies of Anticancer Agents Often Generate Misleading and Clinically Irrelevant Information

### 7.1. The Standard In Vitro Colony Formation Assay Lacks Specificity

Most of us studying cancer cell response to genotoxic stress have been in the same boat! Our own group has been using clonogenic and other cell-based assays for assessing cell death in different cell types after treatment with physical and chemical genotoxic agents for decades (e.g., [158,159,160]), but we soon learned our lesson that we had been misinterpreting the outcome of such assays. It became clear that exposure of solid tumor-derived cell lines (with differing p53 status) to moderate doses of DNA-damaging agents that are typically used in the clonogenic assay triggers proliferation arrest (dormancy) rather than apoptosis or other modes of cell death (reviewed in [31,134,135]). Thus, the clonogenic assay performed with such cell lines primarily determines the ability of a test agent to convert a fraction of the potential colony-forming cells into cells that fail to form colonies in the time span of the assay, but which remain viable and can exhibit metastatic/stemness/tumor repopulating properties. As noted above, the first insight into this aspect of the clonogenic assay (secretion of growth-promoting factors by cancer cells that had lost their colony forming ability under stressful conditions) was reported by Puck and Marcus in 1956 [33].

### 7.2. Multiwell Plate “Viability” and “Cytotoxicity” Assays Also Lack Specificity When Performed with Proliferating Cultures

**Figure 2:**
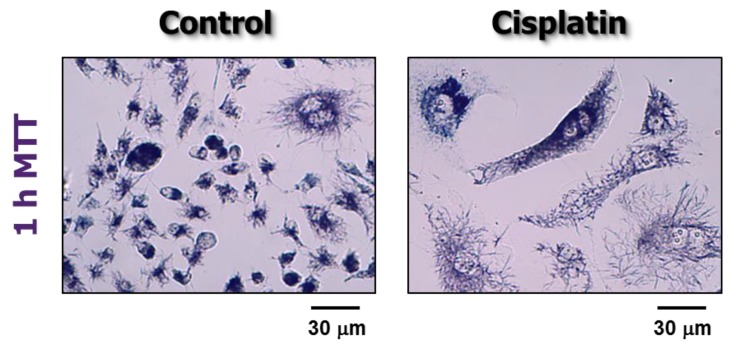
Bright-field microscopy images showing viability of MDA-MB-231 cells before (control) and after incubation with cisplatin (10 µM) for 72 h. Viability was measured by the ability of the cells to convert the 3-(4,5-dimethylthiazol-2-yl)-2,5-diphenyl-tetrazolium bromide (MTT) reagent to its formazan metabolite (dark granules and crystals). Images were acquired after incubation of cells with MTT for ≈1 h. Data reproduced from Mirzayans et al. [30].

Similar to the clonogenic assay, chemosensitivity/radiosensitivity as measured by short-term colorimetric (e.g., tetrazolium-based; crystal violet-based) and fluorometric (resazurin-based, such as CellTiter-Glo) assays primarily reflects proliferation arrest rather than loss of viability (death). This situation pertains to the experimental conditions typically used in most anticancer drug discovery approaches, including the standard NCI screen [3,4,5,6]. This often involves treatment of cells in the exponential phase of growth with a test compound for 48 h, followed by a brief (1–4 h) incubation with a viability-indicator agent [3,4,5,6]. To appreciate the peril in assuming that such assays measure loss of viability/cytotoxicity in proliferating cultures it is important to consider some key experimental details. We will mainly focus our assessment on the chemotherapeutic drug cisplatin and the most widely used indicator agent 3-(4,5-dimethylthiazol-2-yl)-2,5-diphenyl-tetrazolium bromide (MTT). MTT is a yellow tetrazolium salt which is metabolized by viable cells to a purple water-insoluble formazan. MTT metabolites can be visualized as intracellular granules and crystals under a light microscope [32] (also see Figure 2). Crystal violet (gentian violet) is a blue, aniline-derived dye that binds to cellular macromolecules.

In a typical multiwell plate assay, the cells are inoculated at optimal densities in the wells of a microtiter plate and incubated overnight. The medium is then replaced with fresh medium containing cisplatin (treated wells) or the solvent (sham-treated control wells). After incubation for 48 h, the cells are incubated with an indicator agent such as MTT [161] or crystal violet [162]. For the MTT protocol, the cells are incubated with MTT (between 1 and 4 h) to allow its conversion to the purple metabolite by viable cells. A solvent is added to each well to solubilize the purple formazan granules and crystals, and the optical density of the resulting purple color in the medium is determined by a plate reader (wavelength range, 550–600 nm), from which the 50% inhibitory concentration (IC_50_) for cisplatin is calculated [161]. For the crystal violet protocol, the cells are incubated with the crystal violet solution; after extensive washing with water, crystal violet bound to cellular proteins/DNA is then solubilized by incubating the cells with methanol, and this is followed by measurement of the optical density (570 nm) of treated and control wells, from which the IC_50_ values are calculated [162]. What factors contribute to these IC_50_ values?

As shown in Table 1, several factors can contribute to the reduction in the purple color intensity used to calculate the IC_50_ values, including (i) a decrease in the metabolic activity of individual cells (i.e., treated cells are viable, but still convert MTT to its formazan metabolite—albeit at a reduced rate—when compared to sham-treated control cells); (ii) transient cell cycle checkpoint activation, which promotes survival by facilitating DNA repair; (iii) short-term proliferation arrest due to, e.g., reversible apoptosis (anastasis) that might ultimately lead to the emergence of tumor-repopulating cells; (iv) long-term proliferation arrest (dormancy) that may or may not be reversible, but is often not associated with loss of viability; and (v) bona fide loss of viability (death). The last four factors apply globally to all multiwell plate assays irrespective of the type of indicator agent used, whether requiring metabolism (e.g., MTT and other tetrazolium salts; resazurin) or not (e.g., crystal violet, a protein/DNA-binding dye).

### 7.3. Potential Use of Multiwell Plate Assays for Identifying Agents Capable of Killing Dormant Cancer Cells

The use of multiwell plate colorimetric/fluorometric assays (e.g., MTT, CellTiter-Glo, crystal violet) to measure cell viability or cytotoxicity requires that the cells be non-proliferating to avoid complications in data interpretation arising from treatment-induced cytostatic responses. For proliferating cells, the manufacturers of such assay kits do point out the importance of optimizing cell number per well for a given culture, but fail to emphasize the importance of factoring in the effect of proliferation arrest, induced by a test agent, on colorimetric/fluorometric measurements. Perhaps this is due to the misconception that therapy-induced cancer cell proliferation arrest and death are one and the same.

When performed with non-proliferating cells (e.g., cells that have undergone SIPS), however, such assays are anticipated to measure cell viability. As shown in Table 1, the IC_50_ values measured by assays that rely on cell metabolic activity (e.g., MTT, CellTiter-Glo) are expected to reflect (i) reduction in the metabolic activity per cell; and (ii) loss of cell viability (death). On the other hand, the IC_50_ values measured by the crystal violet staining assay are expected to only reflect loss of cell viability.

## 8. Concluding Remarks

As pointed out by Amend et al. [23], the observation that a tumor is comprised of multiple cancer cell sub-populations with different sizes and ploidy (intratumor heterogeneity) was first reported over a century ago (in the mid 1800s) by scientists who were equipped with only a simple (light) microscope, and often with no camera. Unfortunately, the importance of such observations went largely unnoticed by the majority of the scientific community, perhaps because of the development and widespread use of state-of-the-art high-throughput assays that generate averaged results for a population of cells. 

In this article, we provided a brief review of the ever-growing complexity of cancer cell response to genotoxic agents, reflecting in part the tumor-repopulating properties of dormant cancer cells as well as cancer cells exhibiting molecular/cellular features of apoptosis, that were revealed by microscopic evaluation and other single-cell (versus population averaged) observation methods. In light of this complexity, we examined the biological end points measured by popular cell-based assays, namely: (i) the colony formation assay which has been regarded as the gold standard for assessing radiosensitivity and chemosensitivity; and (ii) multiwell plate “viability” and “cytotoxicity” assays that are performed with proliferating cell cultures. The widely held notion that the end point measured by such assays reflects cancer cell death is not supported by a wealth of experimental data obtained by single-cell analysis.

The lesson learned from studying therapy-induced apoptosis is that activation of apoptotic signaling (e.g., caspase activation, PS externalization, nuclear fragmentation, cell shrinkage, and membrane blebbing) in cancer cells does not necessarily lead to their demise, at least in solid tumors. Cancer cells undergoing apoptosis can not only recover from the brink of death through anastasis, but they can also secrete tumor promoting factors which is initiated by caspase-3 and involves the ATM-dependent DNA damage response. Time will tell whether such unexpected survival/oncogenic features of dying cancer cells are limited to apoptosis or will be seen for ferroptosis and other modes of cell death [16].

So why is cancer still the leading cause of death in high income countries? Despite cautionary articles published by the Nomenclature Committee on Cell Death [155] and others [32,156,157] on the potential misinformation perpetuated by preclinical assays, short-term multiwell plate assays continue to be widely used in numerous cancer-related studies reported in high impact factor journals, and the results are often misinterpreted in terms of cell death. Such studies often propose novel therapeutic strategies without considering the complexities of intrinsic and acquired resistance of cancer cells to genotoxic stress, including those outlined in this article (also see Figure 1). As a case in point, tumor repopulation associated with dormant cancer cells (e.g., PGCCs) requires several weeks (if not months) of incubation following treatment with genotoxic agents to be fully manifested, which is far beyond the time span of conventional preclinical assays that are performed with cultured cells and live animals (reviewed in [31]). Furthermore, the impact of cell fusion (cancer cell–cancer cell; cancer cell–leukocyte) on metastasis and tumor repopulation post-therapy is not recapitulated in cell-based assays and may be overlooked in preclinical tumor growth studies using animal models. It is therefore not surprising that despite investing billions of dollars to fight this disease in the past half century, metastatic cancer still remains largely lethal and incurable [2,23].

Unveiling the molecular events that underlie intratumor heterogeneity in terms of therapy resistance together with using preclinical assays under optimal conditions to avoid misinformation, more appropriate experimental design, and rigorous data interpretation, will hopefully lead to clinically relevant strategies for treating the recurrent/metastatic disease that remains a major global health issue. To this end, careful consideration of the end points measured by the various cell-based assays, presented in this article (e.g., Table 1), singled out the multiwell plate crystal violet-staining assay as a potentially useful tool for identifying agents capable of killing dormant cancer cells.

## Figures and Tables

**Table 1:** Genotoxic stress-induced responses that contribute to reduction in colorimetric/fluorometric measurements in multiwell plate assays in proliferating versus non-proliferating cultures.

Assay	Proliferative Status	Responses Contributing to IC_50_ Values
**Tetrazolium-Based Resazurin-Based** (e.g., MTT, MTS, XTT, WST-1, WST-8, CellTitre-Glo)	Proliferating cultures (≥24 h treatment)	- Decrease in metabolic activity of individual cells - Checkpoint activation (proliferation arrest) - Reversible apoptosis (proliferation arrest) - Onset of senescence (proliferation arrest) - Polyploidy/multinucleation (proliferation arrest) - Loss of cell viability (death)
	Non-proliferating cultures	- Decrease in metabolic activity of individual cells - Loss of cell viability (death)
**Crystal Violet Staining**	Proliferating cultures (≥24 h treatment)	- Checkpoint activation (proliferation arrest) - Reversible apoptosis (proliferation arrest) - Onset of senescence (proliferation arrest) - Polyploidy/multinucleation (proliferation arrest) - Loss of cell viability (death)
	Non-proliferating cultures	- Loss of cell viability (death)

## Abbreviations

NCI	National Cancer Institute
EMT	Epithelial to mesenchymal transition
PGCCs	Polyploid giant cancer cells
SIPS	Stress-induced premature senescence
CDK	Cyclin-dependent kinase
ATM	Ataxia telangiectasia mutated
SASP	Senescence-associated secretory phenotype
BRAF	Serine/threonine-protein kinase B-Raf
EGFR	epidermal growth factor receptor
MMR	Mismatch-repair
MOMP	Mitochondrial outer membrane permeabilization
APAF-1	Apoptosis-activating factor-1
DNase	Deoxyribonuclease
PS	Phosphatidylserine
TUNEL	Terminal deoxynucleotidyl transferase dUTP nick end labeling
NF-κB	Nuclear factor kappa B
STAT3	Signal transducer and activator of transcription 3
DFNA5	Deafness, autosomal dominant 5
CRM1	Chromosomal region maintenance 1
MTT	3-(4,5-dimethylthiazol-2-yl)-2,5-diphenyl-tetrazolium bromide
IC_50_	Half-maximum inhibitory concentration
XTT	2,3-bis-(2-methoxy-4-nitro-5-sulfophenyl)-2*H*-tetrazolium-5-carboxanilid
WST-1	Water-soluble tetrazolium salt-1
